# Human–Wildlife Coexistence in Urban Wildlife Management: Insights from Nonlethal Predator Management and Rodenticide Bans

**DOI:** 10.3390/ani10111983

**Published:** 2020-10-28

**Authors:** Christian Hunold, Maz Mazuchowski

**Affiliations:** 1 Department of Politics, Drexel University, 3025 MacAlister Hall, Philadelphia, PA 19104, USA; 2 Department of Biodiversity, Earth and Environmental Science, Drexel University, 2024 MacAlister Hall, Philadelphia, PA 19104, USA; amm665@drexel.edu

**Keywords:** urban wildlife management, human–wildlife coexistence, multispecies flourishing, human–animal studies

## Abstract

**Simple Summary:**

We seek to understand how U.S. cities manage human coexistence with wild animals that are often disliked, specifically coyotes and rats. To this end, we analyze urban wildlife management plans from around the country that propose to strengthen human–wildlife coexistence. Remarkably, some cities are learning to tolerate and even welcome wild predators, such as coyotes, as long as they do not endanger human safety. Killing aggressive individuals remains a management option of last resort. Alternatively, rats are not tolerated at all, and the use of rodenticides to control rat populations remains widespread. Emerging local restrictions on the use of some rodenticides seek to protect the lives of carnivores who feed on rodents. We discuss what the increased popularity of less lethal forms of urban wildlife management can tell us about the capacity of cities to promote the wellbeing not only of people but of wild animals too.

**Abstract:**

Conceptions of human–wildlife coexistence that acknowledge nonhuman wild animals as fellow urban dwellers with legitimate claims on shared urban spaces are starting to influence urban wildlife management practices. Insofar as at least some wild animals have successfully achieved membership in urban society, how has this revaluation affected how urban wildlife is governed? Our interpretive policy analysis explores this question in two areas of urban wildlife management where practices are becoming less lethal: predator management and rodent control. A directed qualitative content analysis of U.S. urban wildlife management plans and rodent control strategies reveals a shift from conflict to coexistence as the basis for understanding human–wildlife relations in urban settings. Indiscriminate killing of urban wildlife is condemned as unethical as well as impractical, and lethal control figures as a measure of last resort that must be rationally justified. Commensal rodents, however, do not benefit from this shift toward coexistence between humans and nonhuman species. Campaigns to restrict the use of rodenticides are intended to protect carnivores, not the rodents themselves. Though urban wildlife management is consistent with some elements of the vision of multispecies flourishing developed by human–animal studies scholars, not all species benefit equally from this transition, and the legitimacy of wild animals’ claims on shared urban spaces often remains contingent on their good behavior.

## 1. Introduction

As U.S. cities continue to invest in green infrastructure, urban environments are becoming more hospitable to wildlife [1]. Yet cities have long been thought to serve primarily human interests, with nonhuman animals (nonhuman animal is the preferred terminology in human–animal studies; hereafter, we use the term animals) often confined to an existence in the shadows of human spaces [2]. As greened cities attract more wild animals, the newcomers tend, over time, to shed their misanthropy and become more visible to human observers. Greater visibility, however, increases the potential for conflicts with humans [3,4]. Moral panics about the unwanted “invaders” abound, along with demands for their removal or, where that is impossible, for restoration of wild animals’ presumed natural fear of humans [5,6]. New imaginaries of urban human–wildlife coexistence are sorely needed, however [3,7,8,9]. To that end, scholars working at the confluence of human–wildlife interactions research and critical animal studies critique anthropocentric conceptions of cities and question the effectiveness as well as the ethics of lethal methods of wildlife management [10,11,12,13,14,15,16]. Efforts to specify what the “good city” might look like in more-than-human terms demand that humans recognize nonhuman animals as fellow urban dwellers with legitimate claims on shared urban spaces, claims that are not provisional, contingent on good behavior [3,9]. Notwithstanding methodological differences among their academic disciplines, philosophers [17,18,19,20], geographers [2,11,21], planners [3,22,23], and conservation scientists [8,24] who theorize multispecies flourishing in urban settings have converged on a distinction between thin and thick conceptions of nonhuman belonging.

Thin conceptions of coexistence consign humans and animals to their respective material and symbolic spheres—to “parallel planes” in Michelfelder’s [19] terminology—that do not, and are not meant to, intersect with one another. This minimalist ethic of coexistence between humans and nonhuman species demands little of humans beyond tolerating urban animals’ presence, provided they primarily inhabit designated parks and other green spaces without making a nuisance of themselves. For the most part, humans go about their lives while animals go about theirs, rarely coming into contact with one another. Thicker conceptions of coexistence ask, more ambitiously, what it means to merge these parallel planes of existence and to invite forms of interspecies sociability “founded upon a more robust sense of belonging than mere toleration or just getting along” [19]. Here, toleration and impatience for boundary violations give way to generosity and hospitality founded upon recognition of mutual entanglement among species. Writing about Australian white ibis who inhabit urban areas, McKiernan and Instone [21] formulate the challenge of thick coexistence as follows: “How do we relate to the ibis outside a dualistic frame of either pest or victim? How do we transform the usual narrative of eradication to a story of entangled human–nonhuman neighborliness?”

A formidable barrier to such a reimagining is the persistence of contradictory beliefs about the place of animals in society [25]. While many urban dwellers are excited about sharing their city with wild animals, others are indifferent, annoyed, or afraid, particularly with regard to predators [26]. Though many charismatic species (e.g., raptors) are admired [27], various abject ones (e.g., rats) number among countless “unloved others” [28,29]. That certain polarizing species (e.g., raccoons) are simultaneously beloved and despised further complicates urban wildlife management [18]. Wildlife management, moreover, is for many cities a low priority, carried out by multiple municipal agencies with conflicting missions and messaging regarding the disposition of wildlife [30,31,32,33]. Moreover, in wildlife management animals have traditionally appeared as objects of expertise and targets of intervention, as populations rather than as individuals, and rarely if ever as fellow inhabitants of multispecies society [12]. Mounting empirical evidence against human exceptionalism is challenging these precepts, however [34]. Social mammals, for example, possess sophisticated systems of communication [14] and employ moral norms of fairness, empathy, trust, and reciprocity that are intelligible to human observers [35,36]. Bats talk [37], and fish need friends [38]. At a minimum, this transition from ethology to ethnology, rooted in scientific and philosophical advances, opens up space for exploring multispecies relationalities that do not treat animals as objects. For example, advocates of “compassionate conservation” seek to nudge wildlife management toward conceptions of coexistence that take animals’ sentience seriously [39,40,41]. We acknowledge that this transition is ongoing, partial, and contested. We also note, however, that incorporating respect for animals’ sentience into wildlife management practices does not depend on settling ontological claims to everyone’s satisfaction [24,33].

Thus, we ask to what extent urban wildlife management practices in U.S. cities reflect emerging conceptions of human–wildlife coexistence that acknowledge wild animals as fellow urban dwellers with legitimate claims on shared urban spaces [3,9]. If at least some wild animals have successfully achieved membership in urban society, has this revaluation affected how urban wildlife is governed? We pursue this question by analyzing urban wildlife management plans that show a commitment to peaceful human–wildlife coexistence, and urban rodent control approaches that restrict the use of second-generation anticoagulant rodenticides. Our interpretive policy analysis of these management discourses reveals three themes of coexistence-based urban wildlife management: nonhuman belonging, lethal management as a last resort, and mutual accommodation. However, the extent to which the presence of these ideas in urban wildlife management indicates a meaningful shift from human–wildlife conflict to peaceful coexistence varies by species and does not, for the most part, extend to commensal rodents.

## 2. Materials and Methods

In order to understand how wildlife management practices in U.S. cities may be incorporating conceptions of multispecies coexistence we analyzed wildlife management and rodent control policy documents produced by municipal governments, fact sheets published by wildlife advocacy organizations, and newspaper articles. Our non-probabilistic sampling of these materials was guided by the study’s aim to understand whether management discourses concerning “unloved others,” such as urban predators and rodents, are showing signs of turning “the usual narrative of eradication to a story of entangled human–nonhuman neighbourliness” [21]. Thus, we selected urban wildlife management plans that foreground nonlethal management of predators (mainly coyotes) and rodent control approaches that prioritize alternatives to second-generation anticoagulant rodenticides. We selected twenty-eight documents for analysis—fourteen each on wildlife management and on rodent control (see Appendix A).

We approached these research materials from the methodological perspective of interpretive policy analysis. Interpretive policy analysis, rooted in a social constructivist worldview of knowledge production, presupposes that problems addressed in policymaking have different meanings for different groups of people [42]. Engagement with these situated meanings, and how those meanings are enacted, lies at the heart of interpretive policy analysis [43]. Thus, we do not ask, for example, whether wildlife management plans assess coyote behavior “right,” based on scientific knowledge produced by wildlife biologists and conservation scientists. Rather, we investigate what coyotes, as rendered in coexistence-friendly urban wildlife management plans, *mean* to these municipalities with respect to the animals’ membership (or non-membership) in urban society. Given our analytic emphasis on meaning-making around human–wildlife coexistence informed by a qualitative research design, our data are not representative of urban wildlife management in the United States as a whole. The study’s focus on the western U.S. simply reflects the reality that coyotes have been present in many western cities for decades, prompting these cities to develop coyote management plans, and that campaigns to ban second-generation anticoagulant rodenticides have been particularly active and successful in California.

Using this qualitative–interpretive perspective, we conducted a directed content analysis of our sample of wildlife management materials [44]. Unlike a conventional content analysis, wherein researchers generate codes directly from the data without the benefit of theory, our directed content analysis was informed by the conceptions of multispecies coexistence and entangled human–animal relationalities discussed in the introduction. These theoretical concepts directed us to examine the data for themes of nonlethal management and of coexistence more generally. The analysis proceeded in three steps. First, each researcher read all of the documents independently and identified preliminary themes related to the research question. We then exchanged our notes and reviewed each other’s interpretation of the materials. Finally, we jointly discussed our coding of themes and thus generated a matrix of themes and subthemes regarding coexistence-based urban wildlife management that we believe best reflects the content of the research materials (Table 1).

## 3. Results

### 3.1. Managing Urban Wildlife for Coexistence

#### 3.1.1. Nonhuman Belonging

The urban wildlife management plans we analyzed argue for the desirability of human–wildlife coexistence. Certain native species—e.g., gophers, opossums—are highlighted for their ecological significance as prey animals or as predators. More generally, the plans take a firm stand against eradicating species humans may find troublesome, such as black-tailed prairie dogs in Denver or raccoons in Chicago. In some parts of the country, acceptance of urban wildlife entails living alongside predators that occasionally injure or even kill people. For example, the City of Boulder acknowledges that black bears and mountain lions inhabit areas of the city that provide resources for them and that “it is not desirable, practical nor feasible to completely eliminate these animals in the urban area” [45]. Rather, the city declares that “Boulder is in mountain lion habitat. Therefore, residents must practice behaviors that accommodate co-existence” [45].

If wild animals, including predators who may pose some risk to humans, cannot and should not be eliminated from cities, how do cities propose to manage human–wildlife coexistence? Urban wildlife management plans concede that insofar as wild animals have made their home in urban areas, humans must learn to live with them—though in cases of serious conflict, the plans caution, human safety and wellbeing trump the interests of wild animals.

Though wild animals are recognized as making valuable contributions to urban ecosystems, they are not necessarily welcome in all parts of the city, only some of which are designated as wildlife habitats. Nor are all species equally welcome—most notably coyotes, native canids increasingly common in cities across the United States who are frequently the focus of citizens’ anxieties about living alongside wild animals. Thus, the City of Davis Coyote Management and Coexistence Plan illustrates what amounts to a zoning code style division of the city into spaces where coyotes belong and where they do not belong:

*Coyotes are considered important members of a healthy ecosystem and should be encouraged to occur in the city’s open space and habitat areas that exist adjacent to, and outside of, the urban limit (e.g., urban/agricultural transition areas and other city owned open space and habitat areas within the planning area). Hazing shall not occur within these areas*.

[46]

On the one hand, the plan declares that coyotes who inhabit land located on the edge of the city are not to be disturbed there. That is no small victory, given that many humans have rather mixed feelings about living alongside coyotes at all. On the other hand, coyotes found in areas of Davis not defined as “agricultural transition areas and other open space habitat” find themselves on shakier ground. While there are no plans to evict the animals from “neighborhood or community spaces,” citizens are asked to take steps to make such areas inhospitable to coyotes, by removing attractants and hazing. Insofar as coyotes cannot be entirely discouraged from populated areas of the city, tolerating their presence there is contingent on the animals’ good behavior, defined as showing aversion, if not fear, of humans and refraining from aggression toward humans and their pets. Humans, in this management scheme, are responsible for “keeping coyotes in their community wild” by teaching coyotes the rules of “active coexistence” based on spatial zones of belonging and not belonging, on the assumption that coyotes—intelligent social mammals—are capable of learning and observing rules of social interaction with humans [46].

Chicago’s Wildlife Management and Coexistence Plan takes a different approach. Here, managers reject zones of spatial separation and policing of habitat boundaries in favor of emphasizing improvement of habitat connectivity as a means to reduce fragmentation and to discourage wildlife from inhabiting “highly residential and densely populated urban areas.” This spatially more fluid conception of nonhuman belonging acknowledges the reality of wild animals moving freely around the city in search of resources and, moreover, does not depend on the widespread participation of citizens in efforts to deter animals from residential areas. Rather, Chicago envisions that urban planning and design can facilitate the movement of wild animals within and between habitats in ways that reduce the potential for human–wildlife conflict [47].

#### 3.1.2. Nonlethal Management

The urban wildlife management plans in our sample reject the indiscriminate killing of urban wild animals on ethical and practical grounds, framing the taking of an animal’s life as an option of last resort that requires rational justification. Thus, Denver’s coyote management plan emphasizes “preventative measures and nonlethal controls. Lethal measures are taken only as a last resort” [48], as when an identifiable coyote is involved in “fairly serious aggressive interactions with humans” [45]. Culling is not considered an acceptable management strategy for coyotes. Ideally, “only specific animals may be targeted and general culling may not occur since removing a group of territorial coyotes will create an undefended area into which transient coyotes will flow” [49]. Davis, California will not use city funds “to trap and kill coyotes, unless there is a clear and imminent public safety threat” [46].

Though endorsing nonlethal management as the preferred approach signals that wild animals, including native predators such as coyotes, are valued members of the urban community, individual animals remain killable under certain conditions. The decision to take a coyote’s life turns on what counts as evidence of an “individual aggressive coyote,” “fairly serious aggression,” and so on. Here the plans seek to subject management responses, including life-and-death decisions, to transparent criteria for diagnosing abnormal behavior and weighing risks to public safety accordingly. Corresponding to types of behavior and levels of risk is a set of tiered responses including “public education, outreach community meetings, hazing, and lethal control as a last resort if there is a dangerous coyote or a public safety risk” [50]. Generalized fear of predators and threat perceptions associated with the animals’ mere presence in an area and with their normal behaviors are typically addressed with public education and community outreach. At the other end of the spectrum—attacks on humans—a lethal response may be required [50]. Identifying risks associated with encounters that humans experience as ambiguous—e.g., when coyotes patrolling their territory are perceived as following humans—is more challenging. To that end, management plans rely on a decision matrix that combines judgments about the type of coyote behavior and, for lack of a better term, the vibe of an encounter to determine whether a management response is warranted and, if so, which one.

Colorado cities, for example, seek to classify normal, habituated, and dangerous coyote behavior. The presence of coyotes in and coyotes passing through developed areas are considered normal behaviors in Colorado’s Front Range and do not warrant a response, though hazing of coyotes in developed areas is encouraged “to re-instill a fear of humans” [50]. Habituated behaviors are more troubling, as when a coyote’s “behavior exhibits little wariness of the presence of people and the coyote is comfortable passing frequently though developed areas” or when a coyote “frequently predates on unattended cats and dogs or other domesticated animals” [50]. Dangerous behavior occurs when a coyote attacks a human or, short of that, bares teeth, lunges, nips at clothing, or growls at a human(s) and/or poses a significant threat to human safety.

#### 3.1.3. Mutual Accommodation

Strategies to facilitate the mutual accommodation of humans and wild animals in urban spaces focus on normalizing human–wildlife interactions that are typically unproblematic while preventing (or managing) interactions that might endanger public safety. Normalization starts from the assumption that wild animals seek out urban areas for reasons that make sense to them (e.g., availability of shelter and food). Normalization also involves educating citizens about why animals might be attracted to an area and explaining that routine behaviors, such as patrolling territory, pose little risk to humans. In addition to furnishing basic information about wildlife biology and ecology, normalization entails combating unrealistic expectations and dispelling common myths, such as reminders that daytime sightings of raccoons or foxes are perfectly normal and very rarely a cause for alarm. To the contrary, human enjoyment of wildlife is widely cited as one of the reasons for cultivating an urban wildlife habitat in the first place. The Colorado City of Broomfield, for example, seeks “to foster an appreciation and enjoyment of local wildlife” [50]. Perhaps most notably, thriving colonies of Brazilian free-tailed bats in Austin, Texas have acquired the status of a spectacular urban wildlife viewing attraction that is heavily promoted by the city and draws thousands of visitors every year [51].

With respect to occasionally troublesome species such as coyotes or black bears, public education and outreach initiatives invoke notions of “active” or “educated” coexistence that emphasize teaching humans and animals how to behave around one another [45,46,47,48,49,50]. Humans are encouraged or required to remove wildlife attractants from their properties (e.g., picking up fallen fruit, storing trash in bear-resistant containers) and to practice hazing techniques that teach animals to fear humans and thus prevent habituation. Though maintaining parallel planes may be unrealistic in more populated urban areas, the behavioral norms associated with active coexistence are designed to accommodate the needs of humans and nonhuman species by minimizing interspecies contact, or at least the most harmful interactions. Humans and wild animals may inhabit the same spaces, but the goal of management is for their activities to overlap as little as possible in order to minimize the potential for conflicts. Interactions that go beyond observation from a distance are strongly discouraged. The interspecies distancing involved here is not just spatial in nature, but also rules out many typically harmless social interactions between humans and wild animals that might routinely occur in residential areas. Animals in particular are to be discouraged from taking too much of an interest in their human neighbors. In fact, being too friendly toward people is equated with habituation, and may prompt wildlife officials to take action, such as stepping up hazing efforts or community outreach. Policing and maintaining boundaries between humans and wild predators remains the backbone of active coexistence as envisioned by urban wildlife management plans.

### 3.2. Rodent Control and Banning Rodenticides

#### 3.2.1. Nonhuman Belonging

Rats are not welcome in human spaces. Recently, however, there has been a growing shift in thinking about rodenticides, particularly in the western United States [52,53]. Conservation biologists have linked the deaths of many wild predators, including mountain lions, cougars, coyotes, owls and other birds of prey, to second-generation rodenticides through biomagnification [54,55]. While carnivores do not consume rodenticides from bait boxes directly, they consume the rodents who have ingested the poison, thus poisoning themselves. Second-generation anticoagulant rodenticides do not take effect immediately, meaning rats can repeatedly return to the bait box and ingest significantly more than one fatal dose of poison before death occurs. There have also been thousands of reported cases of poisons affecting pets and children [56,57]. The movement to ban rodenticides to prevent unintentional poisonings began in California. In 2014, the state restricted the sale and use of second-generation anticoagulant rodenticides to licensed exterminators and to farmers [58]. Malibu, Moorpark, Thousand Oaks and other California cities expanded these restrictions and stopped using anticoagulant rodenticides in city-owned spaces [59]. In September 2020, the State of California banned the sale and use of second-generation anticoagulant rodenticides, with few exceptions. There has been much backlash and disagreement over the decisions to move away from rodenticides, as rats remain prevalent in many urban areas. Known to live in unsanitary spaces and carry diseases such as Methicillin-resistant Staphylococcus aureus (MRSA), typhus, and hantavirus, among others, many see the presence of rats as a threat to public health [60,61]. Many city officials do not believe that it is feasible to stop killing rats, especially in areas with persistent rat problems. Officials recognize, however, that there are many effective alternative methods of rodent control, including snap traps, improving sanitation, and exclusion measures that prevent rodents from entering a property in the first place [57,62].

Policies to exclude rats from human spaces can be linked to the generally low tolerance levels society has towards the species. Many other species, such as birds and squirrels, are welcome urban dwellers. To say that rats are not granted the same consideration of multispecies belonging in urban environments would be an understatement. There is very little tolerance for coexisting with rats in shared urban spaces [60,62]. The behaviors and characteristics of rats do not align with the expected behaviors and characteristics humans place on their wild neighbors [61]. Rats are not tolerated, and even mere signs of their activity, such as droppings or burrows, are met with disdain, fear, and anger [62,63]. The reality that rats are one of the most abundant species of wildlife found in urban areas does not temper the abjection that evidence of their presence provokes in many humans.

Rats have been associated with disease for thousands of years, and this association has trained humans to try to eradicate rats at all costs to avoid disease [61]. That rat populations bounce back quickly in the wake of eradication campaigns goes more or less unacknowledged [63]. Increased sightings of rats in parks and other outdoor spaces create moral panics. Urban rat infestations are regular fodder for local and national news, with spikes in sightings creating public relations nightmares for sanitation departments, health departments, visitor bureaus, and elected officials [60]. Even as increasingly more studies demonstrate the intelligence and emotional range of rats, empathy with rats remains in short supply and their status as pest animals is not up for debate [61]. Support for the idea that rats belong in cities is absent from the anti-rodenticide campaign materials we have analyzed. Though ever present, rats never belong in human spaces.

#### 3.2.2. Nonlethal Management

In September 2020, California became the first U.S. state to ban second-generation anticoagulant rodenticides. The law provides for some exemptions (e.g., medical facilities, slaughterhouses), but second-generation anticoagulant rodenticides may no longer be used in restaurants, grocery stores, homes, housing facilities, schools, and office buildings [64]. Rats may still be killed in many other ways, using snap traps, carbon dioxide asphyxiation, and first-generation rodenticides. Non-toxic alternatives, however, are also part of the debate about how best to control rat populations.

Suggested nonlethal alternatives to rodenticides focus on prevention of rodent infestations before they can occur. Exclusion, the practice of ensuring that any and all possible entrances to a property are sealed to prevent rats from entering the building, is one of the most frequently recommended nonlethal solutions [59,61,65,66]. Another is to improve sanitation in urban areas in order to reduce rats’ access to food waste [60,61,66]. Ensuring trash is stored securely and nearby areas remain clean from food waste and potential shelters deters rats from occupying these spaces. Rats are intelligent and highly adaptive to environmental changes [61]. When humans create an environment that allows rats to thrive, rats will very quickly adjust to the conditions and make themselves at home. Following this logic, creating a less hospitable environment by disposing of food properly and securing trash until it is time to be picked up should discourage rats from inhabiting an area [61]. However, integrated pest management—combining non-toxic techniques such as sanitation, rodent-proofing, etc.—for commensal rodents is not well-studied, and existing evidence is mixed [67].

Most of the nonlethal methods of control are preventative measures to reduce the likelihood that rats will be attracted to certain locations. The one nonlethal method that can be utilized after rats have moved into an area is the application of contraceptives to reduce fertility in future generations [61,63]. Most nonlethal methods, however, are not very effective if rats are already occupying the locations where they are not wanted, which is when people tend to notice them and search for ways to eliminate of them. People tend to not think about the existence of rats until they notice the signs of their presence, by which time it is too late for preventive measures. This is when they turn to lethal methods to eliminate them, which are widely available and socially acceptable [67]. Thus, controlling rat populations still mostly involves killing rats.

Raptors Are The Solution (RATS), a California-based advocacy organization, is campaigning for the use of birds of prey as a natural and ecologically sound lethal solution to rodent infestations. Raptors naturally prey on rats, and encouraging owls and hawks to live in urban areas near rodent populations can reduce the number of rats without the need for poison. RATS encourages the use of owl nesting boxes to lure these raptors to the area for natural pest control as they are efficient killers of rats [54]. The use of birds of prey as rodent management promotes a solution that is not only non-toxic, but also benefits the urban ecology by stimulating the circle of life. Rodents are still killed through this method, but not at the hands of humans, and to the mutual benefit of raptors and humans. Not only does the endorsement of raptors provide a solution for rodent management, but humans also benefit from seeing desirable wildlife in their neighborhoods they might not see otherwise.

#### 3.2.3. Mutual Accommodation

Rodenticides and other uses of lethal control may not be the perfect solutions for dealing with urban rats, but they do not require much human input or effort [67]. Many of the nonlethal alternatives do, a fact that proponents of non-toxic alternatives both acknowledge and seek to address. Rats, adaptive as they are, thrive by learning from human behaviors [61]. For example, if humans continuously leave trash in an accessible place such as an unsecured dumpster, rats will learn that that dumpster will be a steady source of food. In turn, humans can learn to modify their behaviors to discourage rats from occupying spaces where they are unwanted. Humans can learn how to properly exclude rats from buildings, how to store trash to deny rats access, and how to safely manage rats that have bypassed their efforts and entered their property without harming other wildlife or risking the safety of pets and children [56,65,66]. Sanitation and exclusion deter rats from entering human-occupied spaces in the first place, and a focus on these methods can aid in reducing the amount of poison that enters the ecosystem. Educating people by showing them that they have the power to control rat populations by making adjustments to their own behavior can pave the way for both humans and rats to learn to live in urban environments together with minimal conflict.

Our reading of municipal rodenticide bans and campaign materials shows, however, that the form of peaceful coexistence being contemplated here is very thin. Mutual accommodation with urban rats seems to depend, on the one hand, on recognition of the inevitability of their presence and, on the other hand, on their exclusion, to the extent possible, from spaces where they pose actual risks to human health. Cities are not always the cleanest locations, which allows rats to thrive by feeding on trash and hiding in every nook and cranny. It is simply not possible to eliminate every source of food and shelter for rats in cities, and therefore, rats are not going to leave anytime soon [60,62]. Despite their inevitable presence, however, people actually do not come into conflict with urban rats until they notice signs of their occupancy, such as droppings or chew marks [61]. People tend not to care about rats’ general existence so long as they do not become visible. Therefore, the key to normalizing rats as urban wildlife may be to ensure they remain invisible. This can be accomplished by teaching humans to do their part to prevent and deter rats from residing in an area where they would thrive, reproduce, and become visible. For the time being, an uneasy truce based on parallel planes of existence may be the best commensal rodents can hope for.

## 4. Discussion

Our results indicate that urban wildlife management practices are tentatively shifting toward coexistence, though not for all species. This shift also falls short of single plane conceptions of multispecies flourishing. Remarkably, some cities are learning to tolerate and even welcome wild predators, such as coyotes. Alternatively, rats are barely tolerated, if at all. Moreover, the conceptions of nonhuman belonging that animate urban wildlife management discourse remain indebted to the conflict prevention management paradigm, and thus attached primarily to thin rather than thick conceptions of multispecies coexistence. On the one hand, certain species are believed to be capable of learning how to be good neighbors to humans, provided humans are prepared to uphold certain rules of engagement. On the other hand, very little in the way of actual interspecies entanglement is tolerated in the wildlife management materials we have analyzed. Even with species welcome as fellow urban dwellers, policing intra-urban habitat boundaries and attending to conflict prevention remain central pillars of managing for what is termed “active” or “educated” coexistence. Humans are encouraged to appreciate the wild animals with whom they share yards, streets, and neighborhoods, both for their beauty and for their contribution to biodiversity—but only from a distance. More intimate forms of interspecies sociability, such as provisioning or play, are emphatically discouraged. If there is human–nonhuman neighborliness here, it is contingent, at risk whenever humans’ and animals’ indifference and wariness toward the other begins to waver. The consequences of trespassing onto one another’s plane of existence are not shared equally by humans and nonhuman species, however. Showing too much aggression can be just as dangerous to an animal’s wellbeing as being too friendly, an impossible bind that mirrors the dynamics of social exclusion and othering experienced by marginalized human communities [68]. These systems of power and injustice have attracted increased scrutiny from theorists of multispecies justice [69].

However, we found some recognition in these wildlife management practices of more entangled multispecies relationalities, particularly an emerging acceptance of wild animals’ apparent disregard for intra-urban habitat boundaries. Simply put, some urban wildlife management plans divide the city into suitable and unsuitable wildlife habitats. The reality is less clear-cut, of course, in that the spatially complex tapestry of sparsely developed wildlands, neighborhood parks, cemeteries, golf courses, road verges, railroad corridors, and residential yards found in many cities provides perfectly good habitats for all sorts of wild animals. Chicago’s conception of nonhuman belonging retains the idea that residential areas are inappropriate as wildlife habitats because they multiply risks for humans and animals alike. However, the city’s commitment to improving urban green spaces to make these areas more accessible to animals via the construction of green infrastructure such as wildlife corridors at least gestures at a more equitable sharing of urban spaces by humans and wild animals. Such more realistic assessments of the fluid nature of how nonhuman species actually inhabit urban spaces are a key step toward relinquishing the idea that urban wild animals ought somehow to be confined to designated green spaces and, thus, toward embracing thick conceptions of multispecies coexistence.

Alongside messaging that culling urban coyotes is counterproductive from a risk management perspective, typologies of coyote behaviors seek to subject management interventions to rational examination. The narrative of eradication may have faded from view, but individual animals may still be killed under certain conditions. Coyotes, for example, may be killed following incidents of serious aggression, such as attacks on humans (and on leashed pets), but they may not be killed simply for being coyotes. Surely this makes a difference. Granted, attempts to label various coyote behaviors as normal, habituated, and dangerous are inevitably reductive and do not always offer a clear-cut answer in specific instances of conflict, e.g., as when a coyote charges a dog while defending a den site. However, such typologies place many routine coyote behaviors squarely beyond the reach of management interventions, including some that many people may find disturbing, such as coyotes making themselves visible when patrolling their territories or coyotes preying on free-roaming cats.

Few if any of these considerations are afforded to urban rats. We found few affirmations of belonging, nonlethality, and mutual accommodation concerning commensal rodents. While coyotes, black bears, and even mountain lions—though certainly polarizing—are increasingly welcome as urban dwellers, rats are not. It is difficult even to conceive of ways of living peaceably alongside rats that are predicated on tolerating—let alone enhancing—rats’ visibility, ideas that quite simply do not figure in the rodenticide ban campaign materials and city websites we have analyzed. Other coexistence campaigns are moving away from culling, as less lethal options to manage human–wildlife relations are explored. Rats, however, remain undesirable wild neighbors and their lives disposable. Even if rodenticide bans were to become more widespread, other methods of lethal control would remain available. Managing the animals out of existence is no less an option for rats than it is for coyotes, however. Rats are incredibly intelligent and attuned to human habits, and these traits mean they will continuously adapt to changing urban environments and never completely disappear despite humans’ best efforts [61,62]. Perhaps, then, if rats were to be accepted as unavoidable inhabitants of the urban ecosystem, their presence might trigger fewer attempts to poison them and, indirectly, the predators who prey on rats. The RATS campaign arguably at least points in that direction insofar as harnessing raptors as nature’s pest control brigade normalizes rats as ecologically valuable prey animals.

## 5. Conclusions

The evolution of thick and thin conceptions of nonhuman belonging in urban wildlife management varies depending on which species are sharing urban spaces with humans. Some charismatic species are in the process of thickening their membership in urban societies to the point where they enjoy full access to some urban spaces, and provisional access to others. While these species are adjusting to living alongside humans, humans are learning how to coexist with their new neighbors. Urban wild animals are still expected to abide by the rules and boundaries set by people, but even for species as polarizing as coyotes mere tolerance is giving way to something like an invitation to share designated green spaces with humans [70]. Rats, on the other hand, are barely touched even by thin conceptions of belonging in human spaces. Rather, rats do not belong in urban spaces at all, even in those locations where other wild animals are usually allowed to thrive, such as public parks. For the time being, rats remain firmly encoded as pests to be eradicated, albeit by ecologically more benign methods.

That said, few people who came of age in the industrial cities of the mid-20th century could have imagined that these same cities would in just a few decades attract species of wildlife that then inhabited primarily rural landscapes, beyond the walls of the polis. The blurring of human–wildlife boundaries is a work-in-progress, with no self-evident endpoint. Not all species are welcome in cities, but those that are have enhanced not only urban biodiversity but also the meaning of cities. If nothing else, deemphasizing lethal force to regulate urban wildlife populations concedes that cities are not exclusively human spaces, but (however provisionally) spaces for wild animals too. As the size and the diversity of urban wildlife populations continue to grow, a shift toward thicker conceptions of nonhuman belonging that deemphasizes the policing of spatial boundaries between human and nonhuman spaces in favor of recognizing manifold human–nonhuman entanglements in shared spaces is certainly desirable. There is no guarantee, of course, that such new imaginaries of coexistence will prevail, that narratives of eradication will keep being transformed into stories of neighborliness, least of all for those “unloved others” for whom even mere toleration remains presently out of reach. The dynamics of the urban multispecies society emerging from the push and pull of fear and acceptance, of resentment and hospitality merits further research. To that end, proposals to replace the conflict-prevention paradigm with a conflict-to-coexistence continuum that is less quick to cast wild animals as troublemakers who bear the brunt of responsibility for negative human–wildlife interactions represent a promising step toward thicker of multispecies flourishing [71].

## Figures and Tables

**Table 1:** Coexistence in urban wildlife management.

Themes	Subthemes
Nonhuman belonging	Conditional on spatial and behavioral criteria (toleration; selective empathy) Unconditional (hospitality; expansive empathy) Native/non-native status
Lethal control as last resort	Criteria of killability (species; behavior) Nonlethal strategies (design, teaching and learning, human behavior) Rodenticide alternatives mostly lethal
Mutual accommodation	Human–wildlife interactions Normalization Learning Coexistence Conflict management Visibility/invisibility

## Appendix A

Urban Wildlife Management Documents:
Project Coyote. Dogs and coyotes. Available online: http://www.projectcoyote.org/wp-content/uploads/2016/08/Dogs_Coyotes.pdf (accessed on 1 October 2020).
Project Coyote. Informational brochure. Available online: http://www.projectcoyote.org/wp-content/uploads/2015/02/PC_brochure.pdf (accessed on 1 October 2020).
Project Coyote. Coexisting with coyotes brochure. Available online: http://www.projectcoyote.org/wp-content/uploads/2015/10/Coexisting_Brochure_oct2015.pdf (accessed on 1 October 2020).
Project Coyote. Denver’s coyotes learn to live with human neighbors. Available online: http://www.projectcoyote.org/foxdenver.pdf (accessed on 1 October 2020).
Merlin Tuttle’s Bat Conservation. Brazilian free-tailed bats—Congress Avenue Bridge bat colony. Available online: https://www.austinbats.org/bats (accessed on 1 October 2020).
Austin Bat Refuge. Don’t blame bats. Available online: https://austinbatrefuge.org/covid-19/# (accessed on 1 October 2020).
Stafford Mader, L. Coexisting with the coyotes of Travis County: Austin’s wildlife management program goes countywide. Available online: https://www.austinchronicle.com/news/2019-02-22/coexisting-with-the-coyotes-of-travis-county/ (accessed on 1 October 2020).
City of Boulder, CO. Urban wildlife management plan. Available online: https://bouldercolorado.gov/wildlife/urban-wildlife-management-plan (accessed on 1 October 2020).
City of Denver, CO. Avoiding conflicts with wildlife in the city. Available online: https://www.denvergov.org/content/dam/denvergov/Portals/747/documents/Natural_Areas/wildlife/wildlife-conflict-info.pdf (accessed on 1 October 2020).
City of Denver, CO. Coyote management plan. Available online: https://www.mspca.org/wp-content/uploads/2015/08/denver-coyote-management-plan.pdf (accessed on 1 October 2020).
City and County of Broomfield, CO. Coexistence with wildlife policy. Available online: https://www.broomfield.org/DocumentCenter/View/1392/CoexistencePolicyFinal?bidId= (accessed on 1 October 2020).
City of Davis, CA. Coyote management and coexistence plan. Available online: https://www.cityofdavis.org/home/showdocument?id=2896 (accessed on 1 October 2020).
City of Chicago, IL. Wildlife management and coexistence plan. Available online: https://www.chicago.gov/content/dam/city/depts/cacc/PDFiles/Chicago_WIldlife_Plan_FINAL_8.9.19.pdf (accessed on 1 October 2020).
City of Lake Forest Park, WA. Accepted wildlife management plan. Available online: https://www.cityoflfp.com/DocumentCenter/View/487/Wildlife-Management-Plan---4-6-12-3?bidId= (accessed on 1 October 2020).
Rodent Control Documents
California Department of Fish and Wildlife. Rodenticides. Available online: https://wildlife.ca.gov/Living-with-Wildlife/Rodenticides (accessed on 1 October 2020).
City of Davis, CA. Rodenticide Hazards. Available online: https://www.cityofdavis.org/city-hall/urban-wildlife/rodenticide-hazards (accessed on 1 October 2020).
Cudmore, B. A New Approach to Oregon’s Growing Rat Problem. Available online: https://www.nrdc.org/stories/new-approach-oregons-growing-rat-problem (accessed on 1 October 2020).
Malibu, California. Rodent Control & the Environment. Available online: https://www.malibucity.org/750/Rodent-Control-the-Environment (accessed on 1 October 2020).
McFadden, D. Pest Control: Exclusion Is the Most Powerful Weapon. Available online: https://www.foodqualityandsafety.com/article/exclusion-pest-control/?singlepage=1 (accessed on 1 October 2020).
Moorpark, CA. Anticoagulant rodenticides. Available online: http://www.moorparkca.gov/579/Anticoagulant-Rodenticides (accessed on 1 October 2020).
Parsons, M. H., & Munshi-South, J. Better rat control in cities starts by changing human behavior. Available online: https://theconversation.com/better-rat-control-in-cities-starts-by-changing-human-behavior-129232 (accessed on 1 October 2020).
Poison Free Malibu. Rodents. Available online: https://poisonfreemalibu.org/rodents/ (accessed on 1 October 2020).
Raptors Are The Solution. RATS Fact Sheet. Available online: https://www.raptorsarethesolution.org/wp-content/uploads/2016/10/RATS-fact-sheet.pdf (accessed on 1 October 2020).
Raptors Are The Solution. RATS Brochure. Available online: https://www.raptorsarethesolution.org/wp-content/uploads/2018/06/RATS_brochure_2018_HomePrint.pdf (accessed on 1 October 2020).
Richardson, J. Super rats or sickly rodents? Our war against urban rats could be leading to swift evolutionary changes. Available online: https://theconversation.com/super-rats-or-sickly-rodents-our-war-against-urban-rats-could-be-leading-to-swift-evolutionary-changes-125902 (accessed on 1 October 2020).
Sabalow, R. & Reese, P. As rats overrun California cities, state moves to ban powerful pest-killers. Available online: https://www.sacbee.com/news/local/environment/article232687952.html (accessed on 1 October 2020).
Serieys, L. Why Do Poisons Matter? Available online: http://www.urbancarnivores.com/poisons/ (accessed on 1 October 2020).
Williams, T. Poisons Used to Kill Rodents Have Safer Alternatives. Available online: https://www.audubon.org/magazine/january-february-2013/poisons-used-kill-rodents-have-safer (accessed on 1 October 2020).
